# Increasing hay inclusion in silage-based receiving diets and its effects on performance and energy utilization in newly weaned beef steers

**DOI:** 10.1093/tas/txaa026

**Published:** 2020-03-03

**Authors:** Dathan T Smerchek, Elizabeth M Buckhaus, Katie D Miller, Zachary K Smith

**Affiliations:** Department of Animal Science, South Dakota State University, Brookings, SD

**Keywords:** corn silage, grass hay, naïve calves, net energy

## Abstract

The influence of grass hay (GH) inclusion in replacement of corn silage in receiving diets on growth performance and dietary net energy (NE) utilization was evaluated in newly weaned beef steers (*n* = 162 Charolais-Red Angus cross steers; initial body weight **[**BW] = 278 ± 13.4 kg). Treatments were (DM basis): 1) 0% GH, 2) 10% GH, or 3) 20% GH inclusion in replacement of corn silage in receiving diets fed to newly weaned beef steers for 56 d. The study was conducted from October to December of 2019. Data were analyzed as randomized complete block design with pen serving as the experimental unit for all analyses. Increasing dietary inclusion of hay had no influence (*P* ≥ 0.11) on final BW, ADG, gain:feed or observed/expected dietary NE_M_ and NE_G_, observed/expected dry matter intake (DMI), or observed/expected ADG. GH inclusion increased (linear effect, *P* = 0.01) DMI. Observed DMI for all treatments was approximately 15% to 17% less than anticipated based upon steer growth performance and tabular NE values. Evaluation of observed/expected ADG was 31% to 37% greater than expected for the steers in the present study. Particles less than 4 mm increased (linear effect, *P* = 0.01) and greater than 4 mm decreased (linear effect, *P* = 0.01) as GH replaced corn silage in the receiving diet. As the proportion of particles greater than 4 mm increased, cumulative ADG was decreased. These data indicate that GH should be considered in corn silage-based receiving diets to improve DMI. In high-risk calves, improved DMI could result in a lesser incidence of morbidity, although no morbidity was observed in any steers from the present study.

## INTRODUCTION

The period that new cattle are received following weaning and transportation to the feedlot is a critical time in beef cattle production. A primary challenge during this receiving phase is the stress of: weaning, transportation, lack of feed and water, and introduction to unfamiliar feed resources (Loerch and Fluharty, 1999; Blom, 2019). Feed intake of newly received feedlot cattle can range from 1% of body weight (BW) in morbid calves to 1.6% of BW in healthy calves (Hutcheson and Cole, 1986). Thus, dry matter intake (DMI) of newly received cattle is often managed in accordance with set protocols developed by the consulting nutritionist or veterinarian and feed yard managers. This is to ensure cattle are consuming feed above maintenance as quickly as possible post-arrival to the feed yard in order to minimize morbidity and reduced animal growth performance. Preston (2007) indicated that in lighter weight calves, the addition of roughage to receiving calve diets might not be beneficial since the calves are at an inadequate DMI level. Preston (2007) postulated that offering newly weaned calves a more energy-dense diet with a lower roughage content may help in achieving the energy demands of the beef calve at a lower DMI. In the most recent feedlot nutritionist survey, only 4.2% of respondents indicated that they use corn silage as a primary roughage source in receiving calf diets (Samuelson et al., 2016). However, corn silage is a primary feed ingredient for beef production in the Midwest. It is a readily digestible energy and neutral detergent fiber (NDF) source and is an option for marketing home-raised feedstuffs through cattle. The sources of dietary roughage in receiving diets fed to feedlot cattle are important in facilitating adaptation to the new diet in naïve, newly weaned feeder calves. Dry forage feedstuffs are more familiar to cattle transitioning into the feedlot from pasture; however, many feedlots in the upper Midwest region of the United States use ensiled forages. A primary deterrent to the use of ensiled feed for naïve calves is that it is an unfamiliar feedstuff to calves coming off of pasture (Blom, 2019). The objective of the present study was to evaluate the influence of increasing levels of dietary grass hay (GH) inclusion to corn silage-based receiving diets on animal growth performance and efficiency of dietary net energy (NE) utilization in newly weaned beef steers.

## MATERIALS AND METHODS

Animal care and handling procedures used in this study were approved by the South Dakota State University Animal Care and Use Committee (Approval Number: 19-054E).

### Animal Management and Dietary Treatments

One hundred and sixty-two, newly weaned, Charolais × Red Angus beef steers (278 ± 13.4 kg) were transported 513 km from a sale barn in western South Dakota to the Ruminant Nutrition Center (RNC) in Brookings, SD, in October 2019. Upon arrival to the RNC, steers were housed in 7.62 × 7.62 m concrete surface pens with 7.62 m of linear bunk-space and provided ad libitum access to long-stem GH (6.18% crude protein, 39.50% NDF, 30.22% acid detergent fiber (ADF), and 4.58% ash) and water. The following day (day −1), all steers were individually weighed (readability 0.454 kg), applied a unique identification ear tag, vaccinated for viral respiratory pathogens: infectious bovine rhinotracheitis (IBR), bovine viral diarrhea (BVD) 1 and 2, parainfluenza3 virus (PI_3_), and bovine respiratory syncytial virus (BRSV) (Bovi-Shield Gold 5, Zoetis, Parsippany, NJ) and clostridials (Ultrabac 7/Somubac, Zoetis). The afternoon following initial processing, all steers were allotted to their study pens (*n* = 9 steers per pen and 6 pens per treatment). The following morning (day 1) all steers were again individually weighed as well as administered pour-on moxidectin (Cydectin, Bayer, Shawnee Mission, KS) according to label directions, and test diets were initiated. On study day 14, all steers were implanted with 200 mg progesterone and 20 mg estradiol benzoate (Synovex-S, Zoetis), and an implant retention check occurred on day 42. The initial on test BW was the average of processing BW (day −1 BW) and day 1 BW. Steers were used to evaluate the effect of GH inclusion in corn silage-based diets on feedlot receiving phase growth performance and efficiency of dietary NE utilization. Test diets were offered on top of long-stem GH for the first 2 d of the receiving period. Treatments consisted of corn silage-based growing diets that included (DM basis): 1) 0% GH, 2) 10% GH, or 3) 20% GH inclusion in replacement of corn silage (Table 1). Diets were fortified with vitamins and minerals to meet or exceed nutrient requirements and provided monensin sodium (DM basis) at 27.6 g/T (NASEM, 2016). There was no morbidity or mortality noted in the present study. Fresh feed was manufactured twice daily in a stationary mixer (2.35 m^3^; readability 0.454 kg). Orts were collected, weighed, and dried in a forced air oven at 100 °C for 24 h in order to determine DM content if carryover feed spoiled or was present on weigh days. If carryover feed was present on weigh days, the residual feed was removed prior to the collection of BW measurements. The DMI of each pen was adjusted to reflect the total DM delivered to each pen after subtracting the quantity of dry orts for each interim period. Actual diet formulation and nutrient composition based upon weekly feed analyses [Crude protein (CP), AOAC (1984); NDF and ADF, (Goering and Soest, 1970); ash and DM, (AOAC, 1990)] and corresponding feed batching records were generated. Diets presented in Table 1 are actual DM diet composition, actual nutrient concentrations, and tabular energy values (Preston, 2016).

**Table 1.** Composition of experimental receiving diets (DM basis)^*a*^

	GH Inclusion, % (DM basis)		
Item	0	10	20
Corn silage^*b*^	73.64	63.67	53.77
Dried distillers grains plus solubles	20.36	20.33	20.29
Grass hay^*c*^	0.00	10.00	19.94
Pelleted Supplement^*d*^	6.00	6.00	6.00
* Soybean Meal*	*(3.936)^*d*^*	*(3.778)^*d*^*	*(3.618)^*d*^*
* Soybean hulls*	*(0.582)^*d*^*	*(0.740)^*d*^*	*(0.900)^*d*^*
* Trace mineralized salt*	*(0.300)^*d*^*	*(0.300)^*d*^*	*(0.300)^*d*^*
* Calcium Carbonate*	*(1.110)^*d*^*	*(1.110)^*d*^*	*(1.110)* ^*d*^
* Premix* ^*e*^	*(0.072)^*d*^*	*(0.072)^*d*^*	*(0.072)^*d*^*
Nutrient composition^*f*^			
* *Dry Matter, %	38.81	41.77	45.38
* *NE_M_, Mcal/kg	1.78	1.74	1.70
* *NE_G_, Mcal/kg	1.16	1.11	1.08
* *Crude protein, %	13.11	13.08	13.09
* *NDF, %	37.09	39.82	43.10
* *ADF, %	26.21	28.08	30.21
* *ASH, %	6.07	6.31	6.48

^*a*^All values except dry matter on a DM basis.

^*b*^Corn silage (*n* = 9 samples) contained (DM basis, except for dry matter): 31.50% dry matter, 6.18% crude protein, 39.50% NDF, 30.22% ADF, and 4.58% ash.

^*c*^Grass hay (*n* = 9 samples) contained (DM basis, except for dry matter): 86.33% dry matter, 7.23% crude protein, 65.50% NDF, 49.94% ADF, and 7.27% ash.

^*d*^Inclusion to total diet DM included in parentheses.

^*e*^Vitamin premix contained (in each 907-kg of supplement): 7,204 g of SBM, 1,972 g of Rumensin-90 (Elanco, Indianapolis, IN), 48 g of vitamin A (650,000 IU/g), 750 g of vitamin E (500 IU/g), 721 g of IntelliBond Zn (Micronutrients, Indianapolis, IN), and 195 g IntelliBond Cu (Micronutrients) for 0% GH; 7,123 g of SBM, 2,022 g of Rumensin-90 (Elanco), 49 g of vitamin A (650,000 IU/g), 769 g of vitamin E (500 IU/g), 726 g of IntelliBond Zn (Micronutrients), and 201 g IntelliBond Cu (Micronutrients) for 10% GH; 7,226 g of SBM, 1,980 g of Rumensin-90 (Elanco), 48 g of vitamin A (650,000 IU/g), 753 g of vitamin E (500 IU/g), 699 g of IntelliBond Zn (Micronutrients), and 184 g IntelliBond Cu (Micronutrients) for 20% GH.

^*f*^Tabular NE from (Preston, 2016) and actual nutrient compositions from weekly assay of individual dietary ingredients and feed batching records.

### Growth Performance Calculations

Steers were individually weighed on days −1, 1, 14, 28, 42, and 56. Weight gain was based upon initial un-shrunk on test BW (average of days −1 and 1 BW) and day 56 BW that was pencil shrunk 4% to account for gastrointestinal tract fill. Daily energy gain (**EG**, Mcal/d) was calculated according to the large frame steer calf equation: EG = 0.0493W^0.75^ × ADG^1.097^ (NRC, 1984). EG was the daily deposited energy and W was the average BW from the 56-d receiving period using initial un-shrunk BW and day 56 BW shrunk 4% (NRC, 1984, 1996). Maintenance energy (**EM**, Mcal/d) was calculated as: EM = 0.077W^0.75^ (Lofgreen and Garrett, 1968; NASEM, 2016). Using the estimates required for maintenance and gain, the performance adjusted (pa) NE_M_ and NE_G_ values, Owens and Hicks (2019), of the diet were generated using the quadratic formula: $x=\frac{-b\pm \sqrt{b^{2}-4ac}}{2c}$, where x = diet NE_M_, Mcal/kg, a = −0.41EM, b = 0.877EM + 0.41DMI + EG, c = −0.877DMI, and NE_G_ was determined from: 0.877NE_M_ −0.41 (Zinn and Shen, 1998; Zinn et al., 2008). Expected DMI (kg/d) was estimated according to the following equation: expected DMI = (0.0493W^0.75^ × ADG^1.097^/tNE_G_) + (0.077W^0.75^/tNE_M_), where tNE_G_ and tNE_M_ are the tabular NE values of the diet based upon formulation [(Preston, 2016); Table 1]. Expected ADG (kg/d) was determined from feed available for maintenance (FFM), feed available for gain (FFG), retained energy (RE; Mcal/d), and W, where FFM = EM/tNE_M_, FFG = DMI − FFM, and RE = FFG × tNE_G_ according to the following equation: expected ADG = (15.54 × RE^0.9116^ × W^−0.6837^).

### Total Mixed Ration Particle Size Distribution

Total mixed ration (TMR) samples were collected once a week (*n* = 7 wk) from each pen in the present study (n = 6 pens per treatment) for a total of 42 replications per treatment. The TMR samples were separated using the Penn State Particle Separator (PSPS) using the methods described by (Kononoff et al., 2003).

### Statistical Analysis

All data were analyzed as a randomized complete block design experiment using the GLIMMIX procedure of SAS 9.4 (SAS Inst. Inc., Cary, NC), considering dietary treatment as a fixed effect, pen location for block, and pen served as the experimental unit for all analyses. Treatment effects were evaluated by the use of orthogonal polynomials (Steel and Torrie, 1960). An *a* of 0.05 determined significance and an *a* of 0.06 to 0.10 was considered a tendency.

## RESULTS AND DISCUSSION

### Animal Growth Performance

Limited work in regard to dry roughage inclusion in receiving diets for healthy beef steers has been conducted (Preston, 2007). Much of the work has been in relation to dietary roughage inclusion as a potential ingredient to dilute energy density of the receiving diet (Galyean and Hubbert, 2014) and has been conducted in high-risk receiving cattle (Rivera et al., 2005). Dietary treatment effects on steer growth performance are presented in Table 2. There was no morbidity or mortality recorded during the course of the 56-d receiving period. Increasing dietary inclusion of hay in corn silage-based receiving diets had no appreciable influence (*P* ≥ 0.11) on final BW, ADG, gain:feed or observed/expected dietary NE_M_ and NE_G_, observed/expected DMI, or observed/expected ADG. GH inclusion in replacement of corn silage in receiving diets increased (linear effect, *P* = 0.01) DMI by nearly 9% for 20% GH compared with 0% GH. Tomczak et al. (2019) noted a 10% increase in DMI for steers offered a roughage-based receiving diet compared with a concentrate diet offered over top of GH fed at 0.5% of BW (DM basis) during a 56-d receiving period and a nearly 10% improvement in ADG. It was also noted that steers offered a roughage-based receiving diet compared with a finishing diet offered on top of GH exhibited greater rumination time for each kg of DMI on days 4, 7, and 12 of the feedlot receiving phase (Tomczak et al., 2019). Although rumination time was not measured in the present study, greater rumination time could potentially offer a myriad of benefits, namely improved ruminal health and greater digestibility of dietary DM.

**Table 2.** Influence of GH inclusion in replacement of corn silage on animal growth performance and dietary energetics of newly weaned beef steers during the feedlot receiving phase

	GH Inclusion, % (DM basis)				*P*-value	
Item	0	10	20	SEM	Linear	Quadratic
Days	56	56	56	—	—	—
Pen, *n*	6	6	6	—	—	—
Steers, *n*	54	54	54	—	—	—
Growth performance^*a*^						
Initial BW, kg	278	278	277	0.3	0.12	0.30
Final BW, kg	352	353	357	2.7	0.21	0.62
ADG, kg	1.33	1.35	1.43	0.048	0.16	0.54
DMI, kg/d	6.46	6.74	7.04	0.105	0.01	0.93
Gain:feed	0.206	0.200	0.204	0.0045	0.72	0.37
Expected DMI, kg	7.60	7.92	8.51	0.208	0.01	0.62
Expected ADG, kg	1.00	1.03	1.05	0.023	0.21	0.91
pa NE, Mcal/kg^*b*^						
Maintenance	2.05	1.99	1.99	0.022	0.10	0.30
Gain	1.39	1.33	1.34	0.020	0.10	0.30
Observed/expected						
NE_M_	1.16	1.14	1.17	0.013	0.45	0.23
NE_G_	1.19	1.20	1.24	0.017	0.11	0.60
DMI	0.85	0.85	0.83	0.011	0.19	0.42
ADG	1.32	1.31	1.37	0.026	0.26	0.35

^*a*^Initial BW was the average of day −1 and day 1 BW, and final BW was from day 56 and was pencil shrunk 4% to account for gastrointestinal tract fill.

^*b*^paNE calculated from observed steer growth performance (Zinn and Shen, 1998; Zinn et al., 2008).

There was a tendency (linear effect, *P* ≤ 0.10) for increasing inclusion of GH to decrease paNE_M and G_. However, this was expected as the GH had lower tabular NE_M_ and NE_G_ values than the corn silage it replaced in the diet (Preston, 2016). Interestingly, observed DMI for all treatments was approximately 15% to 17 % less than expected based upon steer growth performance and tabular NE values, suggesting that high-growth potential steers that exhibit no obvious signs of clinical morbidity do not match model estimates for expected intake and exhibit improved gain efficiency. Additionally, observed ADG was 31% to 37% greater compared with expected ADG when using the large frame steer equation (NRC, 1984) for live weight gain (LWG), suggesting that the growth potential of the steers used in the present study was greater than the estimates for gain when using the LWG equation for large framed steer calves (NRC, 1984).

### Total Mixed Ration Particle Size Distribution and Effects on Cumulative ADG

**Figure 1.**
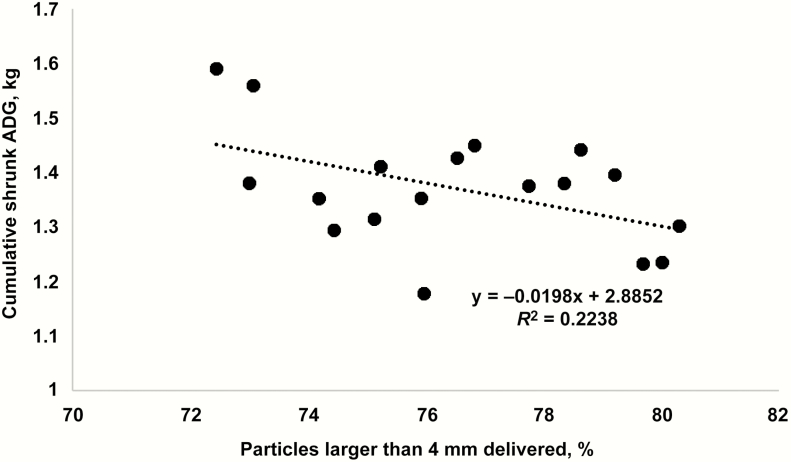
Effect of the proportion of particles greater than 4 mm delivered on cumulative ADG (kg/d). Cumulative ADG = −0.0198 (proportion of particles greater than 4 mm) + 2.8852; *R*^2^ = 0.2238.

The effect of GH inclusion on TMR particle size distribution is presented in Table 3. The corn silage was estimated to have a grain content of greater than 50%. Corn particles were observed on the upper sieves (larger than 4 mm) of the particle separator and would have influenced the proportion of larger particles measured in the present study. It is unknown whether or not the influence of receiving diet on larger particles was an artifact of corn, roughage, or both as the mechanical influence of forage processing is drastically different for corn silage and GH. As GH increased in the receiving diet, there was an increase (linear effect, *P* = 0.01) in the large particles greater than 19 mm. Conversely, as GH increased in the receiving diet, there was a decrease (linear effect, *P* = 0.01) in medium-sized particles from 8 to 19 mm. There was a decrease (quadratic effect, *P* = 0.01) in small particles from 4 to 8 mm in size as GH increased in the receiving diet, being greatest for the 0% GH level and similar for the 10% and 20% GH inclusion diets. Overall, particles less than 4 mm increased (linear effect, *P* = 0.01) and greater than 4 mm decreased (linear effect, *P* = 0.01) as GH replaced corn silage in the receiving diet. Effect of the proportion of particles greater than 4 mm delivered on cumulative ADG (kg/d) was determined (Figure 1). As the proportion of particles greater than 4 mm increased, cumulative ADG was decreased, this could be related to differences in DMI as proportion of larger particles delivered decreased, and this is similar to what others have determined (Blom, 2019). This effect of particle size on observed ADG could be due to a variety of factors such as increased ruminal fill that influenced daily DMI in addition to altered rate of passage that resulted in reduced digestibility of diet DM, although neither of these variables were measured in the present study.

**Table 3.** Influence of GH inclusion in replacement of corn silage on particle size distribution of TMR from newly weaned beef steers during the feedlot receiving phase^*a*^

	GH inclusion, % (DM basis)				*P*-value	
Item	0	10	20	SEM	Linear	Quadratic
Replicates, *n*	7	7	7	—	—	—
Pens, *n*	6	6	6	—	—	—
TMR, % (as-is basis)						
Large (≥19 mm)	6.4	11.9	16.3	0.27	0.01	0.15
Medium (8 to 19 mm)	61.6	54.1	47.7	0.36	0.01	0.23
Small (4 to 8 mm)	11.4	10.3	9.8	0.07	0.01	0.01
Less than 4 mm	20.6	23.8	26.2	0.27	0.01	0.30
Greater than 4 mm	79.4	76.2	73.8	0.27	0.01	0.30

^*a*^Determined according to the study of Kononoff et al. (2003).

## CONCLUSIONS

Steers in the present study had exceptional DMI, ADG, and gain efficiency. This is likely a function of healthy steers that exhibited a great deal of lean growth potential and as such were very efficient on a high-roughage diet. Increasing GH inclusion in replacement of corn silage resulted in improved DMI. As the proportion of particles greater than 4 mm increases, cumulative ADG is decreased. Measuring the proportion of particles larger than 4 mm could be a useful tool in determining the ADG during the receiving period; however, the practicality of use might be limited as it does not incorporate differences in dietary NE and DMI. These data indicate that GH should be considered in corn silage-based receiving diets to improve DMI. In high-risk calves, improved DMI could result in a reduced incidence of morbidity, although no morbidity was observed in any steers from the present study.
